# Impact of COVID-19 infections and vaccination on menstrual cycle symptoms in the south of Jordan: a cross-sectional study

**DOI:** 10.1186/s42506-024-00153-z

**Published:** 2024-04-10

**Authors:** Mohammad A. Abu-Lubad, Munir A. Abu-Helalah, Mohammad S. QawaQzeh, Esra’a F. Alahmad, Malak M. Al-Tamimi, Mohammad K. Ruba’I, Sahm H. Etoom, Rawan K. Alfayoumi, Fatima E. Aldaoudeyeh, Ekram A. AlAbabneh, Ahlam M. Al-kharabsheh, Hussam Alshraideh

**Affiliations:** 1 Department of Microbiology and Pathology, Faculty of Medicine, Mutah University, P.O. Box 7 Mutah, Al-Karak, 61710 Jordan; 2 Department of Family and Community Medicine, School of Medicine, University of Jordan, Public Health Institute, Amman, Jordan; 3 Medical Students at Mutah University, Faculty of Medicine, Al-Karak, Jordan; 4 Department of Obstetrics and Gynecology Faculty of Medicine, Mutah University, Al-Karak, Jordan; 5 Department of Industrial Engineering, College of Engineering, American University of Sharjah, Sharjah, United Arab Emirates

**Keywords:** Jordan, COVID-19, Menstrual cycle, COVID-19 vaccine

## Abstract

**Background:**

Several recent studies have highlighted the need for more evaluation of the impact of COVID-19 infections and vaccines on the reproductive system and menstruation. This study aimed to assess the impact of COVID-19 infection and vaccines on menstrual symptoms.

**Methods:**

A cross-sectional survey utilizing face-to-face interviews from January 1 to 31 March 2022 was conducted in the city of Al-Karak in southern Jordan. The questionnaire included sociodemographic characteristics, medical and reproductive history, the contraceptive method used if any, menstrual cycle (MC) status, previous medical and drug history, and the impact of infection and vaccination on the MC.

**Results:**

The study questionnaire was completed by 400 participants with a mean age of 32.1±12.6 years. Regarding the history of COVID-19 infections, 33.8% of the participants reported a history of confirmed COVID-19 infections, 77.8% of them did not report any menstrual changes following the infection, while the remaining 22.2% reported changes in menstruation. The most commonly reported post-COVID-19 manifestations were irregular (27.6%) and light menstrual cycle (MC) (24.15) or dysmenorrhea (24.1%). Heavy menstruation was reported by 17.2% of participants post-COVID-19 infection. Two-thirds of the study participants (66.6%) reported no changes in the MC following the administration of the COVID-19 vaccine. The most reported symptoms for those who experienced changes in the MC following the vaccination were irregular cycle (13.1%), heavy menstruation (7%), and light menstruation (7%). Other reported symptoms were dysmenorrhea (4.6%), intermenstrual bleeding (1.2%), and amenorrhea (0.5%).

**Conclusion:**

This study revealed minor changes in the MC following COVID-19 infections and administration of the COVID-19 vaccine. These findings are consistent with published reports. It is recommended that future clinical trials for new vaccines for women of childbearing age include outcomes related to sex hormones and MC. Women should be encouraged to take the vaccines and report symptoms to healthcare professionals for further assessment.

## Introduction

The menstrual cycle (MC) is a regular sloughing of the uterine lining regulated by hormones released from the hypothalamic-pituitary-gonadal axis [1]. The length of the MC is the time between the menstrual bleeding and the onset of bleeding in the next cycle [2, 3]. The normal duration of MC is between 25-30 days with a duration of less than 21 days known as polymenorrhagia and greater than 35 days termed as Oligomenorrhe [2, 3]. The typical volume of lost blood during the MC is about 30 ml with volumes greater than 80 ml considered abnormal. Globally, 5% to 35.6% of MCs are irregular during childbearing age [4]. Different stressors implicated in deregulating the MC include certain medications, certain viral infections, structural abnormalities, obesity, sudden weight loss, smoking, and psychological problems [5, 6]. Certain viral infections as Coronavirus (CoV) have been added as an exceptional stressor affecting MC irrespective of whether the individuals have been infected or not, due to the fear of contracting the virus [7]. Changes in menstrual symptoms could be related to the viral infection with SARS-CoV-2 or due to the changes in the stress level associated with the infection state [8]. Infection with SARS-CoV2 affects the immune system and causes an increase in interleukin (IL)-6, IL-8, tumor necrosis factor-α (TNF-α), and other cytokines. This is in addition to the potential alteration of the hypothalamic–pituitary–gonadal axis due to this infection [9, 10].

COVID-19 is the causative agent of the severe acute respiratory syndrome coronavirus 2 (SARS-CoV-2) [11]. COVID-19-associated disorders range from mild to fatal ones [12]. Compared to other countries, thousands of COVID-19-associated deaths in Jordan were reported in addition to those who died without being diagnosed with COVID-19 infection [13].

Different vaccination predicates have been found to play an important role in the adherence of people to the vaccination programs including age, socioeconomic status, education and health literacy [14]. Moreover, the trust in the health environment through proper broadcasting of health information plays an important role in linking people’s information reception and their vaccination intention [15].

MC disorders have a substantial negative impact on quality of life and can have a huge socioeconomic burden for women and the health services [16]. Unfortunately, COVID-19 studies, particularly vaccine trials, ignored its impact on menstruation in the context of how many women have shown MC disorders, how long these persisted, and the impact of these disorders [16].

It has been shown that COVID-19 uses the angiotensin-converting enzyme 2 (ACE2) as a cellular entry receptor [17]. This receptor is highly prevalent in the female reproductive system including the uterus [18]. The binding of COVID-19 to the ACE2 receptor in the endometrium may be associated with the deregulation of the MC [19]. After vaccination, a percentage of women experienced menstrual irregularities such as heavy menstrual bleeding (menorrhagia), frequent bleeding (metrorrhagia/ polymenorrhea), or postmenopausal bleeding [20, 21]. Recently, studies emphasized the relationship between COVID-19 infection and menstrual disturbances [22–24]. There has been a limited number of published studies on the impact of COVID-19 vaccine and COVID-19 infections on menstrual symptoms. Published studies have reported ranges of MC disturbances following COVID-19 vaccination [25–27]. The thousands of self-reported MC related issues were sent to the United States Vaccine Adverse Event Reporting System (VAERS) [28]. The United Kingdom’s Medicines and Healthcare Products Regulatory Agency (MHRA) yellow card surveillance scheme highlights a strong association between MC changes and COVID-19 vaccination [25].

The COVID-19 vaccination program in Jordan started early in January 2020. The most common rerecorded side effects (SE) of these vaccines among Jordanian people include pain, swelling and redness at the site of injection, fatigue, chills, fever, myalgias, headache, and nausea [29]. One possible side effect that needs further investigation is the impact of the vaccine on the MC. Some studies in Jordan have shown that COVID-19 infection could affect the MC [30, 31]. Moreover, it was reported that the timing of MC was altered after vaccination among Jordanian females as shown by another report from Jordan, which concluded that the impact of COVID-19 vaccine on the reproductive system among Jordanian females needs further studies [32]. We conducted this cross-sectional study to investigate the impact of COVID-19 infections and vaccines on menstrual symptoms in Al-Karak governorate.

## Methods

### Study design and sample

This was a cross-sectional survey utilizing structured face-to-face interviews from January first to 31 March 2022. According to national statistics, it is estimated that there are 51519 females aged 15 to 50 in Al-Karak governorate. A sample size of 382 is needed with a margin of error of 0.05. The sample size was obtained through an online sample size calculator provided by Raosoft Incorporation (http://www.raosoft.com/samplesize.html). The selected sample was 400 Jordanian females from the general population. We followed a multistage sampling technique from the general population through a door-to-door approach by research coordinators. Al-Karak governorate was stratified into 3 districts and the sample was obtained from high, middle, and low socioeconomic areas in each district. Villages in Al-Karak were handled as a cluster sample, two villages were selected randomly. In each area, samples were obtained randomly.

Eight final year mixed medical students and internship physicians who received proper training from the principal investigator and the co-investigators collected the data. Permission was granted from the ethics committee in the faculty of medicine at Mutah University under the approval number 1002020 before conducting this study. All questionnaires were completed without including names or other identifiers to maintain confidentiality.

### Inclusion and exclusion criteria

The inclusion criteria were: Jordanian females residing in the selected area for at least 2 years and should have a regular menstrual cycle for one year before receiving the COVID-19 vaccination. The exclusion criteria included females who had prior history of amenorrhea, bleeding disorders, females who underwent abdominal or pelvic surgery, taking any medications that interfere with the normal MC, breastfeeding women, and those diagnosed with a medical condition that could influence cycle regularity, such as endometriosis, polycystic ovarian syndrome, and thyroid disorders. In addition, only women younger than 50 were included and participants should not have a prior history of amenorrhoea.

### Measures

A questionnaire consisting of fifteen questions was developed in English language by a group of experts in the field and based on the literature related to the topic of this research [33–35]. Questions were asked in Arabic by medical students who got training on how to fill in the questionnaire. The questionnaire was divided into three sections; the first section addressed the sociodemographic and medical history of the participants. The second section covered COVID-19 infections and vaccinations while the third section covered menstrual symptoms. The questionnaire was piloted with 30 participants and modified accordingly. Pilot data was not included in the final analysis.

The participants were asked about their background characteristics, including age, marital status, and if married they were asked about the parity and the number of abortions. The married participants were asked about using contraceptive methods including natural method (Breast feeding, coitus interruptus), oral contraception, intrauterine contraceptive device, implant, and injectable progesterone and estrogen. The age of participants at menarche was included in the questionnaire.

An important question included was inquiring about the MC status including regular (21-35 days, 3-8 days length, average bleeding, primary dysmenorrhea), irregular (< 21 days or 35 days), and if they are menopausal. Previous medical history also considered if they are medically fit, or have diabetes, hypertension, thyroid disorders, polycystic ovarian syndrome, and endometriosis. Drug intake history included the use of anticoagulants (aspirin or heparin) and thyroxin as these drugs are known to affect the duration of menstruation. Whether the participants had a history of COVID-19 infection or not was also listed in the questions. The symptoms that the participants experienced during the period after the infection and during the period after receiving the vaccine included heavy MC, intermenstrual bleeding, irregular MC, amenorrhea, dysmenorrhea, light MC, post-menopausal bleeding, and no symptoms at all. Data on vaccination included the kind of vaccine, the number of doses, the date when the participants received the vaccine and the intervals (between less than one month to more than four months ago). Participants’ history of visiting a gynecologist for MC related complaint was included in the questionnaire as well.

### Data analysis

The numbers obtained were expressed as percentages or as mean ± SD. The results were interpreted after tabularizing them in frequencies and percentages. All calculations were carried out using the Statistical Package for the Social Science (SPSS); SPSS Inc., Chicago, IL, USA, version 20.0 for Microsoft Windows. The dependent variable is the symptoms during the period after the infection, while the remaining variables were considered independent.

The key outcomes were the presence of menstrual symptoms and the list of symptoms, if present. No composite symptoms score was calculated. We had no issues with missing data as all interviews were done in a face-to-face manner.

## Results

The study questionnaire was completed by 400 participants with a mean age of 32.1 ± 12.6 years. The average parity was 4 ± 2.5 and abortion 0.8 ± 1.6. The mean age at menarche is 13.4 ± 1.4 years.

As shown in Table 1, half of the study participants were singles. Two thirds of the participants reported that they never used any sort of contraception, while 16.5% of the participants used the natural method of avoidance of fertility days. On the other hand, hormonal contraceptive methods and intrauterine contraceptive device (IUCDs) were used by 9.8% and 9.3% of the participants respectively.

**Table 1:** Background information of study participants of Jordanian women, AL-Karak, 2022

Attribute	Frequency	%
**Marital Status**		
Single	213	**51.4**
Married	189	**45.7**
Divorced/Widow	12	**2.9**
**Contraception method used**		
Hormonal	19	**9.8**
IUCDs	18	**9.3**
Natural Method	32	**16.5**
None	125	**64.4**
**Menstruation status**		
Irregular	43	**10.4**
Menopause	38	**9.2**
Regular no primary dysmenorrhea	68	**16.4**
Regular primary dysmenorrhea	265	**64**
**Medical history**		
History of chronic diseases	66	**16**
Medically free	347	**84**
**Use of Anticoagulants**		
No	400	**96.6**
Yes	14	**3.4**
**History of COVID-19 Infection**		
Yes, I got the infection	126	**30.4**
No, I did not get infected	288	**69.6**
**Symptoms during the period after the infection (*n* = 29 participants)**		
None	99	**77.3**
Heavy MC	5	**3.9**
Intermenstrual bleeding	1	**17.2**
Irregular MC	8	**3.4**
Dysmenorrhea	7	**27.6**
Light MC	7	**24.1**
Post-menopausal bleeding	1	**24.1**
**COVID-19 vaccine type**		
Pfizer	244	**58.9**
Astrazeneca	7	**1.7**
Sinopharm	162	**39.1**
Sputnik	1	**0.2**

*IUCDs *intrauterine contraceptive devices

Menstruation status prior to COVID-19 infections and vaccines, showed a high rate of regular menstruation with primary dysmenorrhea (64%). The 14 participants, who reported that they were on anticoagulants, were excluded from the analysis.

**Fig. 1:**
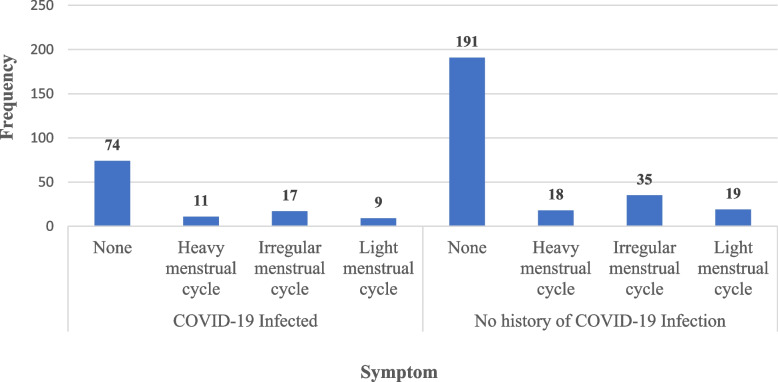
Menstrual symptoms by reported history of COVID-19 infection

Regarding history of COVID-19 infections, 33.8% of the participants reported a history of confirmed COVID-19 infections, 77.8% of them did not report any menstrual change following the infection, while the remaining 22.2% reported changes in menstruation. Figure 1 shows a comparison of menstrual symptoms between participants with a history of COVID-19 and those without a history of COVID-19 infection. There was no statistically significant difference between these two groups. This figure shows that the most commonly reported post-COVID-19 symptoms were irregular MC (8, 3.86%) and light MC (7, 24.15%) or dysmenorrhea (7, 24.1%). Heavy menstruation was reported by 17.2% of participants post-COVID-19 infection.

### Menstrual symptoms post COVID-19 vaccination

All study participants reported that they have received at least one dose of COVID-19 vaccine. Most of them (88.2%) received two doses, while 3 participants only (0.7%) received three doses. The remaining 46 participants (11.1%) received only a single dose of vaccine. Most of the study participants received either Pfizer mRNA Vaccine (58.9%) or the inactivated Sinopharm vaccine (39.1%).

Two thirds of the study participants (66.6%) reported no changes in the MC following COVID-19 vaccine. The most frequently reported symptoms for those who experienced changes in the MC following the vaccination were irregular MC (13.1%), heavy menstruation (7%), and light MC (7%). Other less reported symptoms were dysmenorrhea (4.6%), intermenstrual bleeding (1.2%), and amenorrhoea (0.5%).

Only ten participants sought medical advice from a gynecologist for these symptoms. Outcomes of assessment were abnormal hormonal profile and normal ultrasound for three participants, while two participants had abnormal ultrasound and normal hormonal profile. The remaining five participants had a normal hormonal profile and normal ultrasound.

**Fig. 2:**
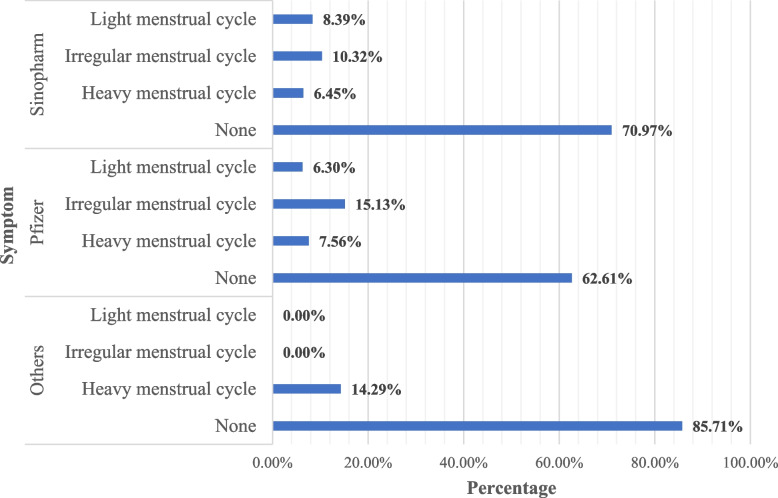
Menstrual symptoms by type of COVID-19 vaccine

Figure 2 shows a comparison of menstrual symptoms by the type of vaccine received by study participants. There was no statistically significant difference between the three groups. The main difference between them is that participants who received other vaccines (other than Pfizer or Sinopharm) did not report irregular MC or light menstruation post-vaccination.

Finally, Table 2 shows a comparison between Sinopharm and Pfizer vaccines in terms of reported symptoms post-vaccination. Overall, more symptoms were reported post-Pfizer vaccine compared with the Sinopharm vaccine. Amenorrhea and intermenstrual bleeding were reported by two and five participants who received the Pfizer vaccine. On the other hand, these two symptoms were not reported by participants who received the Sinopharm vaccine.

**Table 2:** Comparison of self-reported menstrual changes between Sinopharm and Pfizer COVID-19 vaccines

Symptoms	Sinopharm		Pfizer		*P*-value
Irregular MC	Frequency	%	Frequency	%	
Light MC	16	10.3	36	15.1	**0.222**
Heavy MC	13	8.4	15	6.3	**0.5589**
Dysmenorrhea	10	6.5	18	7.6	**0.8274**
Intermenstrual bleeding	6	3.9	13	5.5	**0.6326**
Amenorrhea	0	0	5	2.1	**0.1752**
Post-menopausal bleeding	0	0	2	0.8	**0.6753**
	0	0	0	0	

## Discussion

Several recent studies and reports have highlighted the need for more evaluation of the impact of COVID-19 infections and vaccines on the reproductive system and menstruation. Published data so far are not robust enough to draw a firm conclusion. We conducted this study in Jordan on women of reproductive age from the general population. Our findings revealed overall mild changes in the menstrual symptoms due to COVID-19 infections and vaccines. All participants received COVID-19 vaccines; most received at least two doses. Pfizer and Sinopharm vaccines were the most widely received vaccines. Post-vaccination menstrual changes were also mild with no statistically significant differences between vaccines.

Three quarters of our sample did not report any menstrual change following COVID-19 infection. The most common reported menstrual symptoms for women, who experienced changes in the MC, were irregular MC, light MC, and dysmenorrhea. Heavy menstruation was reported by a small proportion of the study participants. Our results are consistent with the finding of a recent systematic review. This systematic search was conducted to assess the impact of COVID-19 infection on MC. Only three studies were included in the final analysis. Results revealed that SARS-CoV-2 led to menstrual irregularity with decreased menstrual volume and prolonged cycle as the two most commonly reported symptoms [36].

Another study from Ireland included 1031 women of reproductive age who completed an anonymous digital survey as part of an observational study of women’s reproductive health over the course of the pandemic. Around half of the participants (46%) reported a change in their MCs since the beginning of the pandemic. This study revealed significant changes in the MC when compared with the pre-pandemic period [37]. Participants reported more menorrhagia, dysmenorrhea, and a worsening of premenstrual symptoms than before the pandemic. However, this study did not report the proportion of participants who had confirmed COVID-19 infections, and, therefore, the stress level, as described below, could play a major role in the reported symptoms. Another study from China showed results consistent with our findings [22]. This study evaluated the impacts of COVID-19 infection on the sex hormones and menstrual changes among women with confirmed COVID-19 infection. This study revealed that approximately 20% of the patients had a significant decrease in menstrual volume, with no significant correlation to disease severity. Approximately 20% of COVID-19 patients showed prolonged MCs. A smaller proportion of participants reported an increase in menstrual volume and/or MC shortening. Similar finding were reported from Arizona, USA with 16% (*n* = 20) of the participants reporting MC changes following COVID-19 infection [38].

High stress levels due to the infection or the pandemic worldwide are associated with menstrual irregularities. COVID-19 pandemic has changed life worldwide. It has been associated with different stressors such as losing loved ones, losing jobs, and health changes due to the infection [39]. A recent study has revealed a significant prolongation of menses and heavier menstruation among participants with a high perceived stress scale (PSS) compared to those with a moderate COVID-19 PSS [40].

Similar to our findings, a range of MC changes after COVID-19 vaccination have been reported in several studies, including longer and shorter cycles, missed cycles, heavier and lighter menstrual flow, and intermenstrual spotting [37, 41, 42]. The main limitation of the scientific evidence related to COVID-19 vaccines’ impact on reproductive health is that clinical trials of COVID-19 vaccines did not include menstrual changes within their outcomes. The impact of COVID-19 vaccines on MC was assessed in our study based on the participants’ own reporting. Two thirds of study participants reported no changes in the MC following COVID-19 vaccine. The most reported symptoms for those who experienced changes in the MC following the vaccination were irregular MC, heavy menstruation, and light menstruation. Our results are consistent with international reports and published studies. A US cohort provides further evidence of small cycle length changes associated with COVID-19 vaccination and supports our findings [43]. A recently published study from Italy revealed that 50 to 60% of reproductive-age women experienced MC irregularities, regardless of the type of the received COVID-19 vaccine. The incidence of the symptoms was slightly higher (60–70%) following the second dose of the vaccine. The most commonly reported changes were shorter MCs, longer cycles, and heavier menstruation than was expected and usual [44]. The difference in results between our study from Jordan and this Italian study is that around 40% of our sample received the inactivated Sinopharm vaccine while the Italian study population mainly received mRNA vaccines. This could explain the difference in the proportion of reported symptoms.

An important cohort study from the USA showed the waning of menstrual changes in the cycle post-vaccine. This study also revealed that the cycle length changes associated with mRNA vaccines do not appear to differ from those with other vaccine mechanisms. These findings reassure women about the reversibility of menstrual changes post-COVID-19 vaccines [25]. Other factors play a role in MC deregulation among different nations including culture, climate conditions, and sociodemographic characters*.*

### Limitations of the study

The main limitation of our study lies in the differences in intervals post-vaccination between study participants. Additionally, it is based on self-reported changes post-COVID vaccines through retrospective approach subjecting the findings to recall bias. However, our results are consistent with published studies on the impact of COVID-19 infection and vaccines.

## Conclusion

This study revealed minor changes in the MC following COVID-19 infections and administration of the COVID-19 vaccine. There was no statistically significant difference by the history of infection. These findings are consistent with published reports. It is recommended that future clinical trials for new vaccines for women of childbearing age to include outcomes related to sex hormones and MC. Women should be encouraged to take the vaccines and to report symptoms to healthcare professionals for further assessment.

## Data Availability

Data are available from the corresponding author on reasonable request.

## References

[1] Wang C, Pan R, Wan X, Tan Y, Xu L, Ho CS (2020). Immediate psychological responses and associated factors during the initial stage of the 2019 coronavirus disease (COVID-19) epidemic among the general population in China. Int J Environ Res Public Health.

[2] Itriyeva K (2022). The normal menstrual cycle. Curr Probl Pediatr Adolesc Health Care.

[3] Jain M, Gorania N (2015). Abnormal uterine bleeding: a study of menstrual patterns and histopathological patterns in perimenopausal females. Int J Reprod Contracept Obstet Gynecol.

[4] Kwak Y, Kim Y, Baek KA. Prevalence of irregular menstruation according to socioeconomic status: a population-based nationwide cross-sectional study. PLoS ONE. 2019;14(3):e0214071. 10.1371/journal.pone.0214071. 10.1371/journal.pone.0214071 PMC6424400 30889222

[5] Rafique N, Al-Sheikh MH (2018). Prevalence of menstrual problems and their association with psychological stress in young female students studying health sciences. Saudi Med J.

[6] Jung EK, Kim SW, Ock SM, Jung KI, Song CH (2018). Prevalence and related factors of irregular menstrual cycles in Korean women: the 5th Korean National Health and Nutrition Examination Survey (KNHANES-V, 2010–2012). J Psychosom Obstet Gynaecol.

[7] Rodríguez-Hidalgo AJ, Pantaleón Y, Dios I, Falla D (2020). Fear of COVID-19, stress, and anxiety in university undergraduate students: a predictive model for depression. Front Psychol.

[8] Pierce M, Hope H, Ford T, Hatch S, Hotopf M, John A (2020). Mental health before and during the COVID-19 pandemic: a longitudinal probability sample survey of the UK population. Lancet Psychiatry.

[9] Moreno-Perez O, Merino E, Alfayate R, Torregrosa ME, Andres M, Leon-Ramirez JM, et al. Male pituitary–gonadal axis dysfunction in post-acute COVID-19 syndrome—Prevalence and associated factors: a Mediterranean case series. Clin Endocrinol. 2022;96(3):353–62. 10.1111/cen.14537. 10.1111/cen.14537 PMC8444731 34160836

[10] Faraj SS, Jalal PJ (2023). IL1β, IL-6, and TNF-α cytokines cooperate to modulate a complicated medical condition among COVID-19 patients: case-control study. Ann Med Surg.

[11] Umakanthan S, Sahu P, Ranade AV, Bukelo MM, Rao JS, Abrahao-Machado LF (2020). Origin, transmission, diagnosis and management of coronavirus disease 2019 (COVID-19). Postgrad Med J.

[12] Lai C-C, Shih T-P, Ko W-C, Tang H-J, Hsueh P-R. Severe acute respiratory syndrome coronavirus 2 (SARS-CoV-2) and coronavirus disease-2019 (COVID-19): the epidemic and the challenges. Int J Antimicrob Agents. 2020;55(3):105924. 10.1016/j.ijantimicag.2020.105924. 10.1016/j.ijantimicag.2020.105924 PMC7127800 32081636

[13] Khader Y, Al NM (2021). Excess mortality during the COVID-19 pandemic in Jordan: secondary data analysis. JMIR Public Health Surveill.

[14] Umakanthan S, Lawrence S (2022). Predictors of COVID-19 vaccine hesitancy in Germany: a cross-sectional, population-based study. Postgrad Med J.

[15] Umakanthan S, Bukelo M, Bukelo M, Patil S, Subramaniam N, Sharma R (2022). Social environmental predictors of COVID-19 vaccine hesitancy in India: a population-based survey. Vaccines.

[16] Sharp GC, Fraser A, Sawyer G, Kountourides G, Easey KE, Ford G (2022). The COVID-19 pandemic and the menstrual cycle: research gaps and opportunities. Int J Epidemiol.

[17] Wrapp D, Wang N, Corbett KS, Goldsmith JA, Hsieh C-L, Abiona O (2020). Cryo-EM structure of the 2019-nCoV spike in the prefusion conformation. Science.

[18] Dhaundiyal A, Kumari P, Jawalekar SS, Chauhan G, Kalra S, Navik U (2021). Is highly expressed ACE 2 in pregnant women “a curse” in times of COVID-19 pandemic?. Life Sci.

[19] Li X-F, Ahmed A (1996). Expression of angiotensin II and its receptor subtypes in endometrial hyperplasia: a possible role in dysfunctional menstruation. Lab Inves..

[20] Stanton R, To Q, Khalesi S, Williams S, Alley S, Thwaite TV, C. Depression, anxiety and stress during COVID-19: associations with changes in physical activity, sleep, tobacco and alcohol use in Australian adults. Int J Environ Res Public Health. 2020;17(11):4065. 10.3390/ijerph17114065. 10.3390/ijerph17114065 PMC7312903 32517294

[21] Kurdoğlu Z. Do the COVID-19 vaccines cause menstrual irregularities? Int J Womens Health Reprod Sci. 2021;9(3):158–9. 10.15296/ijwhr.2021.29.

[22] Li K, Chen G, Hou H, Liao Q, Chen J, Bai H (2021). Analysis of sex hormones and menstruation in COVID-19 women of child-bearing age. Reprod Biomed Online..

[23] Nazir M, Asghar S, Rathore MA, Shahzad A, Shahid A, Khan AA (2022). Menstrual abnormalities after COVID-19 vaccines: a systematic review. Vacunas.

[24] Mitra A, Verbakel JY, Kasaven LS, Tzafetas M, Grewal K, Jones B (2023). The menstrual cycle and the COVID-19 pandemic. PLoS ONE.

[25] Edelman A, Boniface ER, Male V, Cameron ST, Benhar E, Han L, et al. Association between menstrual cycle length and COVID-19 vaccination: global, retrospective cohort study of prospectively collected data. BMJ Med. 2022;1(1) e000297. 10.1136/bmjmed-2022-000297. 10.1136/bmjmed-2022-000297 PMC9665108 36381261

[26] Alvergne A, Von Woon E, Male V (2022). Effect of COVID-19 vaccination on the timing and flow of menstrual periods in two cohorts. Front Reprod Health.

[27] Lee KM, Junkins EJ, Luo C, Fatima UA, Cox ML, Clancy KB. Investigating trends in those who experience menstrual bleeding changes after SARS-CoV-2 vaccination. Sci Adv. 2022;8(28):eabm7201. 10.1126/sciadv.abm7201. 10.1126/sciadv.abm7201 PMC9286513 35857495

[28] Zhang B, Yu X, Liu J, Liu J, Liu P (2022). COVID-19 vaccine and menstrual conditions in female: data analysis of the Vaccine Adverse Event Reporting System (VAERS). BMC Womens Health.

[29] Omeish H, Najadat A, Al-Azzam S, Tarabin N, Abu Hameed A, Al-Gallab N (2022). Reported COVID-19 vaccines side effects among Jordanian population: a cross sectional study. Hum Vaccin Immunother.

[30] Al-Najjar MAA, Al-Alwany RR, Al-Rshoud FM, Abu-Farha RK, Zawiah M. Menstrual changes following COVID-19 infection: a cross-sectional study from Jordan and Iraq. PLoS ONE. 2022;17(6):e0270537. 10.1371/journal.pone.0270537. 10.1371/journal.pone.0270537 PMC9242447 35767537

[31] Aolymat I (2021). A cross-sectional study of the impact of COVID-19 on domestic violence, menstruation, genital tract health, and contraception use among women in Jordan. Am J Trop Med Hyg..

[32] MM Al-Mehaisen L, A Mahfouz I, Khamaiseh K, N AL-Beitawe S, Al-Kuran OA. Short term effect of Corona virus diseases vaccine on the menstrual cycles. Int J Womens Health. 2022:1385-94 10.2147/IJWH.S376950. 10.2147/IJWH.S376950 PMC9507976 36164386

[33] Takmaz T, Gundogmus I, Okten SB, Gunduz A (2021). The impact of COVID-19-related mental health issues on menstrual cycle characteristics of female healthcare providers. J Obstet Gynaecol Res.

[34] Demir O, Sal H, Comba C (2021). Triangle of COVID, anxiety and menstrual cycle. J Obstet Gynaecol.

[35] Phelan N, Behan LA, Owens L. The impact of the COVID-19 pandemic on women’s reproductive health. Front Endocrinol. 2021:191 10.3389/fendo.2021.642755. 10.3389/fendo.2021.642755 PMC8030584 33841334

[36] Lebar V, Laganà AS, Chiantera V, Kunič T, Lukanović D (2022). The effect of COVID-19 on the menstrual cycle: a systematic review. J Clin Med.

[37] Zechiu I, Gica N, Botezatu R, Peltecu G, Panaitescu AM. Menstrual cycle abnormalities in women vaccinated against COVID-19. Ro J Infect Dis. 2022;25(1) 10.37897/RJID.2022.1.3.

[38] Khan SM, Shilen A, Heslin KM, Ishimwe P, Allen AM, Jacobs ET (2022). SARS-CoV-2 infection and subsequent changes in the menstrual cycle among participants in the Arizona CoVHORT study. Am J Obstet Gynecol.

[39] Nagma S, Kapoor G, Bharti R, Batra A, Aggarwal A (2015). To evaluate the effect of perceived stress on menstrual function. J Clin Diagn Res.

[40] Ozimek N, Velez K, Anvari H, Butler L, Goldman KN, Woitowich NC. Impact of stress on menstrual cyclicity during the Coronavirus disease 2019 pandemic: a survey study. J Womens Health. 2022;31(1):84–90. 10.1089/jwh.2021.0158. 10.1089/jwh.2021.0158 34582731

[41] Alvergne A, Kountourides G, Argentieri A, Agyen L, Rogers N, Knight D (2021). COVID-19 vaccination and menstrual cycle changes: a United Kingdom (UK) retrospective case-control study. iScience.

[42] Male V. Menstrual changes after COVID-19 vaccination. BMJ. 2021; 374 10.1136/bmj.n2211 10.1136/bmj.n2211 34526310

[43] Edelman A, Boniface ER, Benhar E, Han L, Matteson KA, Favaro C, et al. Association between menstrual cycle length and coronavirus disease 2019 (COVID-19) vaccination: a U.S. Cohort. Obstet Gynecol. 2022;139:1–9. 10.1097/AOG.0000000000004695. 10.1097/AOG.0000000000004695 PMC8936155 34991109

[44] Laganà AS, Veronesi G, Ghezzi F, Ferrario MM, Cromi A, Bizzarri M (2022). Evaluation of menstrual irregularities after COVID-19 vaccination: results of the MECOVAC survey. Open Med.

